# Generation of Non-aliased Two-dimensional Acoustic Vortex with Enclosed Metasurface

**DOI:** 10.1038/s41598-020-60836-3

**Published:** 2020-03-02

**Authors:** Jing-jing Liu, Bin Liang, Jing Yang, Jun Yang, Jian-chun Cheng

**Affiliations:** 1 Collaborative Innovation Center of Advanced Microstructures and Key Laboratory of Modern Acoustics, MOE, Institute of Acoustics, Department of Physics, Nanjing University, Nanjing, 210093 P.R. China; 2 Key Laboratory of Noise and Vibration Research, Institute of Acoustics, Chinese Academy of Sciences, Beijing, 100190 P.R. China

**Keywords:** Acoustics, Electronics, photonics and device physics

## Abstract

Two-dimensional (2D) acoustic vortex allows new physics and applications different from three-dimensional counterparts, yet existing mechanisms usually have to rely on active array composed of transducers which may result in complexity, high cost and, in particular, undesired spatial aliasing effect. We propose to generate 2D acoustic vortex inside an enclosed metasurface illuminated by axisymmetric wave carrying no orbital angular momentum. We derive the criterion on unit size for eliminating spatial aliasing effect which is challenging for conventional active approaches and design a membrane-based metasurface to implement our mechanism. The performance of our strategy is demonstrated via precise production of different orders of non-aliased vortices regardless of center-to-center alignment, with undistorted Bessel-like pattern extending to the whole inner region. We anticipate our design with simplicity, compactness, precision and flexibility to open up possibility to design novel vortex devices and find important applications in diverse scenarios such as on-chip particle manipulations.

## Introduction

The past few years witness considerable efforts devoted to both theoretical and experimental study on acoustic vortices due to the fundamental interest and practical importance of their unconventional characteristics such as null pressure amplitude at the core^1^ and spiral phase dislocations^2^. For instance, the orbital angular momentum (OAM) transfer from acoustic vortex beams to matter allows nondestructive and contactless manipulation on tiny objects (e.g. microparticles) and plays an important role such as in biomedical applications^3–12^, and the OAM-based multiplexing and de-multiplexing mechanism offers new route to boost the capacity of acoustic communication^13,14^. In comparison to the three-dimensional counterparts (for which the spiral wavefront extends infinitely alone the propagation direction in three-dimensional space)^15–18^, two-dimensional (2D) acoustic vortices with propagation direction and wavefront in the same plane enable novel in-plane rotational manipulation of individual particle and are of particular significance for lab-on-chip applications and novel flat acoustic functional devices. However, the existing mechanisms for producing 2D acoustic vortices have to rely on active elements which limit their application potential in practice^19–22^. In addition to the complexity and high cost for building complicated array of circularly-arranged and individually-driven transducers, the bulky size of already existing active elements that are usually of wavelength scale^23^ will unavoidably cause spatial aliasing effect^19,24^ in a ring closing to the transducer, which becomes more severe when the centers of vortex and device are not perfectly aligned. Such aliasing effect inevitably shrinks the effective areas of acoustic vortex and impairs the quality of produced spatial pattern, which poses fundamental barriers in precise object manipulation and device miniaturization and hinders their applications such as in on-chip particle manipulation where unnecessary enlargement of acoustic vortex field will lower the manipulation efficiency.

In this paper, we make an attempt to address the above issues by proposing a mechanism for producing 2D non-aliased acoustic vortex (NAAV) inside a geometrical area enclosed by a passive metasurface. We analytically derive the desired distribution of effective parameters along azimuthal angle needed for converting an axisymmetric wave incident from outside into a NAAV and the critical size of each discrete unit cell for minimizing the spatial aliasing effect which is difficult to achieve with active transducers. The reduction in the effective region of produced acoustic vortex field in the presence of aliasing effect is also predicted theoretically. Based on this, a membrane-coated hybrid metamaterial (MCHM) with subwavelength size, full 0-to-2*π* phase modulation and near-unity transmission efficiency is designed as a practical implementation of our mechanism. Compared with traditional active methods, our spatial-aliasing-free 2D vortex production based on metasurface takes advantages of simplicity, fine resolution, high efficiency, compact size and needs no energy supply, with the potential to find application in many diverse fields ranging from on-chip hologram to particle manipulation. We use numerical simulation to verify the effectiveness of our scheme via representative examples of generating NAAV of different topological charge regardless of whether or not the vortex center is aligned with the center of metasurface.

## Results

### The mechanism of generating NAAV with metasurface

**Figure 1:**
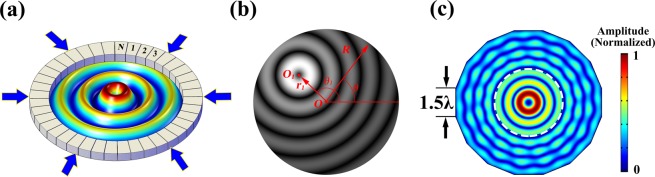
Schematic of the design for generating NAAV. (**a**) Schematic of 2D acoustic vortex generation with metasurface. (**b**) Definition of coordinate systems used in the generation of 2D vortex with a desired *m*th order acoustic vortex centered at (*r*_1_, *θ*_1_). (**c**) Numerical simulation of 2D acoustic vortex generation with *m* = 1, *r*_1_ = 0, *N* = 16 and width of single element equals 1.5*λ*; white dash line denotes the radius of non-aliased region anticipated by Eq. (5).

Figure 1(a) schematically illustrates our proposed mechanism that uses a closed metasurface placed on a circular boundary to azimuthally modulate the propagation phase of a converging cylindrical wave with uniform azimuthal phase and pressure amplitude impinging on it from outside and thereby produce a 2D acoustic vortex within the inner region. First we give analytical derivation of the desired phase profile the metasurface needs to provide, which can be obtained from the acoustic pressure yielded by an outgoing vortex by invoking reciprocity in such a linear acoustic system. For a *m*th order acoustic vortex field centered at (*r*_1_, *θ*_1_) as shown in Fig. 1(b), the acoustic pressure on the boundary is^24^

1 $$p(R,\theta )={p}_{0}\mathop{\sum }\limits_{n=-\infty }^{+\infty }{J}_{m-n}(k{r}_{1}){e}^{i(m-n)({\theta }_{1}-\pi )}{J}_{n}(kR){e}^{in\theta }$$

where ${p}_{0}$ refers to the pressure amplitude, *k* = 2*π*/*λ* is the wave number with *λ* being the wavelength in the background medium (chosen as air here) and *J*_m_(*x*) is a *m*th order Bessel function of first kind. For the cases where the acoustic vortex is centered at origin, one has *r*_1_ = 0 and, by using Graff’s addition theorem, can derive the acoustic pressure and phase profile on the boundary as follows

2 $$p(R,\theta )={p}_{0}{J}_{m}(kR){e}^{im\theta }$$

3 $$\Phi (\theta )=m\theta $$

For a more general situation where acoustic vortex center is not aligned with the origin, i.e., $\,{r}_{1}\ne 0$, one can still avoid simultaneous modulation of both pressure amplitude and phase, and generate a vortex centered at (*r*_1_, *θ*_1_) nearly perfectly by employing the following phase profile

4 $$\Phi {\prime} (\theta )=m\theta +k\sqrt{{R}^{2}+{r}_{1}^{2}-2R{r}_{1}\,\cos (\theta -{\theta }_{1})}$$

Here the off-centered acoustic vortex is realized by introducing an additional phase delay deduced from the distance between vortex center and metasurface center (second term of Eq. (4)), resulting in an illusional circle boundary centered at (*r*_1_, *θ*_1_)^25,26^. Then by adding a phase profile from 0 to 2*mπ* (first term of Eq. (4)), one can generate a 2D vortex with desired center location within the inner region. Considering the finite size of each building block of metasurface, the ideal continuous phase profiles given by Eqs. (3) and (4) must be discretized in practice, which might result in undesired spatial aliasing effect near the boundary and consequent shrinking of the effective region of vortex field as shown in Fig. 1(c). For a metasurface composed of a total of *N* unit cells, the radius *r*_*f*_ of the distortion-free vortex field can be calculated as^24^

5 $${r}_{f}=\lambda (N-m)/\pi e-{r}_{1}$$

Hence, to generate 2D NAAV in a circle region with radius *R*, condition ${r}_{f}\ge R$ is requisite. Substituting $N=2\pi R/H$ and Eq. (5) into this inequity for which *H* is the width of a unit cell, one obtains $H\le H\_{\rm{crit}}=\lambda /e$ where *H*__crit_ is defined as the critical unit size for eliminating the aliasing effect. Notice that this condition is quite difficult, if not impossible, to achieve by using conventional active methods due to both the bulky size of transducers and the complexity and high cost of building a phased array comprising a large number of elements. In stark contrast, our mechanism offers a simple and low cost alternative for generating spatial-aliasing-free 2D vortex field due to the unique subwavelength nature of metasurface unit cell.

### Design and acoustical parameters of proposed MCHM

**Figure 2:**
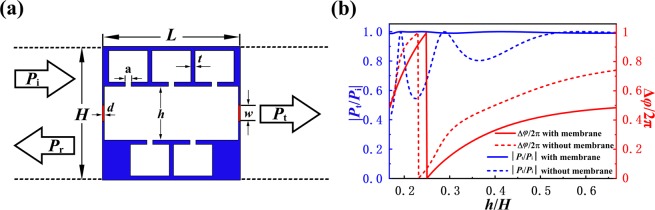
Design and acoustical parameters of MCHM (**a**) 2D cross section view of an individual unit cell of the designed MCHM consisting of a solid hybrid structure (blue part) coated with two thin membranes (red part), with subwavelength scale in all dimensions (*L* = *H* = *λ*/3). (**b**) Simulated phase shift (red) and transmission rate (blue) of MCHM as a function of ratio *h*/*H* provided by such a unit cell with (solid lines) and without (dashed lines) membranes.

In addition to the requirement on the compactness of metasurface unit cell that must not exceed the critical size *H*__crit_, the reproduction of the desired pressure distribution given by Eq. (2) by using passive phase-only elements calls for arbitrary control of abrupt phase shift and zero transmission loss, which is still challenging for the existing designs of metasurface unit cells for modulating the transmitted waves^27–29^. To this end, we propose a design of MCHM satisfying all the above requirements as basic building block of our metasurface. Figure 2(a) schematically depicts the configuration of MCHM which consists of a hybrid solid structure in the middle and two thin membranes coated at both sides. The middle part of MCHM is a straight tube isometrically connecting five resonant cavities with a fixed width *H* and a tunable *h* to span the phase over 2*π* range. According to the acoustic theory applied for such kind of hybrid structure^30^, the effective impedance of the middle part of MCHM suddenly changes near the resonance peaks, which leads to a fluctuant transmission spectrum and substantially deteriorates the quality of the generated 2D vortex field. Hence our design uses the coupling of two resonant membranes at two sides to improve the impedance matching with the background media, resulting in a near-unity transmission efficiency. The underlying physical mechanism is that membranes with resonance frequency being tuned to be identical with the MCHM produce an additional acoustic reactance such that we can compensate for the impedance mismatch in inner structure^31–33^. The structural parameters are chosen as: *H* = *L* = *λ*/3 < *λ*/*e*, *t* = *λ*/100, *a* = 1.25*t*, *w* = 4*t*, and *d* = *t*/8 respectively. Then we numerically simulate the phase shift and transmission coefficient of proposed structure as a function of *h*/*H* and plot typical results in Fig. 2(b). The acoustical parameters of a hybrid structure in the absence of membranes is also calculated for comparison. The numerical results show that such metasurface unit cell provides arbitrary phase shift within the full 2*π* range via adjustment of a single structural parameter while keeping a near-unity transmission efficiency (>0.99) which is ensured by the introduction of membranes (as evidence by the comparison between the cases with and without membranes) and crucial for the high-quality reproduction of target 2D NAAV. It is noteworthy that our mechanism for generating non-aliased vortex in a two-dimensional system is general and can be practically implemented by using various metastructure designs such as some other shell-type metasurfaces^34^. Here we choose to employ our designed MCHM that applies to the production of 2D vortices of different radii by simply controlling a single parameter of unit number *N*, which helps to significantly simplify the design and fabrication of the resulting device as long as the spatial resolution of phase profile is high enough.

### Production of 2D concentric and off-center NAAV

**Figure 3:**
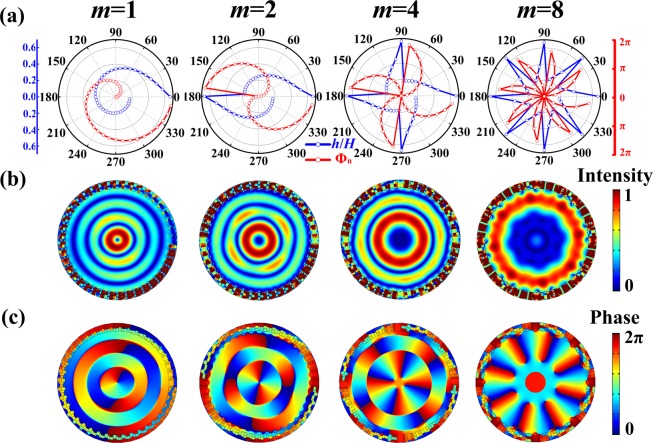
Metasurface for 2D concentric NAAV generation. (**a**) Discrete phase shift profile anticipated by Eq. (6) (red dot line) and corresponding parameter *h*/*H* profile provided by metasurface (blue dot line) for generating NAAV with *m* = 1, 2, 4 and 8. (**b**) Simulated normalized pressure field and (**c**) phase distribution of NAAV with *m* of 1, 2, 4 and 8.

Next we use numerical simulations to demonstrate the capacity of our scheme to generate 2D NAAV by using the metasurface consisting of MCHM and the flexibility of adjusting the topological charge and the location of vortex center. We firstly demonstrate the production of NAAV with center-to-center alignment, i.e., *r*_1_ = 0. Based on the ideal continuous phase profile from Eq. (3), for a metasurface formed by arranging *N* MCHM along azimuthal direction, the discrete phase shift provided by each MCHM is given by

6 $${\Phi }_{n}=(\frac{2m\pi (n-1)}{N})$$

where *n* = 1, 2, 3,…, *N* refers to the *n*th unit cell. In principle, our mechanism can be implemented with fewer units as long as $H\le H\_{\rm{crit}}$. But for 2D vortex, although the reduction of unit number helps to downscale the device size, shrinking of effective region of vortex field would usually be undesired in many practical applications such like particle trapping. Hence, here we set *N* equals 40 such that the metasurface can keep relatively high resolution while avoiding complicated fabrication as far as *m* < 10. For generation of 2D NAAV with four particular topological charges of *m* = 1, 2, 4 and 8, the discrete phase shift profiles predicted by Eq. (6) and corresponding values of *h*/*H* as functions of azimuthal angle are illustrated in Fig. 3(a). Figure 3(b,c) show the simulated normalized acoustic pressure field and phase distribution respectively, which exhibits nearly perfect whole-area 2D acoustic vortex patterns without spatial aliasing effect and shrinking of effective region for all four *m*. Besides, from Fig. 3(c) one can observe the phase discontinuity on the boundaries between two phase rings which stems from the oscillation of Bessel function around zero value. Also, simulation results reveal that the null pressure regions expand with the topological charge increasing and phase change around a circle is 2*mπ*, as expected.

For more general case of producing NAAV whose center can be arbitrarily located within the region enclosed by metasurface (i.e., ${r}_{1}\ne 0$), rewriting Eq. (4) into a discrete form one obtains the phase profile required for each MCHM, as follows

7 $${\Phi {\prime} }_{{n}}=(\frac{2m\pi (n-1)}{N})+k\sqrt{{R}^{2}+{r}_{1}^{2}-2R{r}_{1}\,\cos (\frac{2\pi (n-1)}{N}-{\theta }_{1})}$$

**Figure 4:**
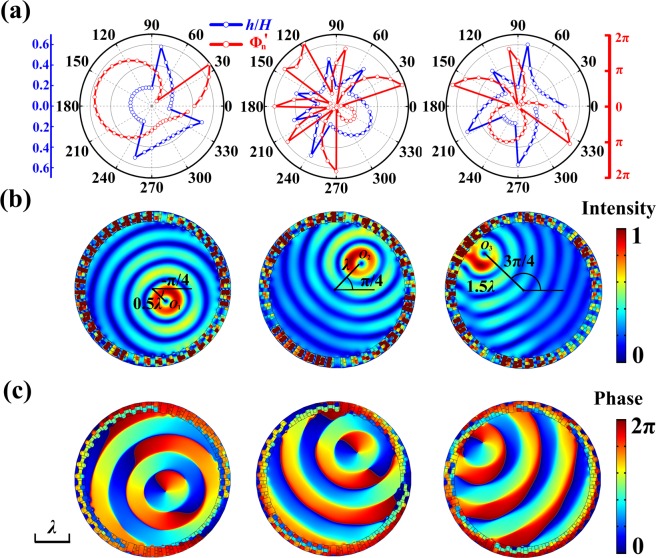
Metasurface for 2D off-center NAAV generation. (**a**) Discrete phase shift profile anticipated by Eq. (7) (red dot line) and corresponding parameter *h*/*H* profile provided by metasurface (blue dot line) for generating first order off-center NAAV centered at *O*_1_, *O*_2_, *O*_3_. (**b**) Normalized acoustic pressure field distribution and (**c**) phase distribution with off-center acoustic vortex centered at *O*_1_, *O*_2_, *O*_3_.

For simplicity without loss of generality, we choose *m* = 1 and *N* = 40 and demonstrate the production of off-centered NAAV with center located at three particular points: *O*_1_ (0.5*λ*, −*π*/4), *O*_2_ (*λ*, *π*/4), and *O*_3_ (1.5*λ*, 3*π*/4) respectively. The discrete phase shift profile given by Eq. (7) and the parameter *h*/*H* are illustrated in Fig. 4(a). Figure 4(b,c) show the normalized acoustic pressure field distribution and phase distribution of the produced acoustic vortex centered at *O*_1_, *O*_2_, *O*_3_. We can observe that our scheme gives rise to the off-centered acoustic vortex precisely at the target location without causing undesired spatial aliasing effect, as evidence by the undistorted Bessel-like pattern extending to the whole inner region. This phenomenon persists when we increase the offset of vortex center significantly.

## Discussion

To conclude, we propose to generate 2D NAAV with enclosed metasurface illuminated by axisymmetric wave carrying no orbital angular momentum. We analytically derive the desired phase profile of metasurface and the criterion on unit size for eliminating spatial aliasing effect which is challenging for conventional active methods. As a practical implementation satisfying these requirements on effective acoustic parameter and physical dimension, we design a MCHM as a basic building block of metasurface which takes advantages of subwavelength scale, arbitrary phase shift within the full 2*π* range and near-unity transmission efficiency. Via two distinct examples of production of 2D concentric and off-center acoustic vortices, we have numerically verified the effectiveness of our scheme for precisely generating 2D NAAV of different orders regardless of center-to-center alignment. In addition, the undistorted Bessel-like pattern generated by our scheme has been extended to the whole inner region, which is difficult, if not impossible, to implement with traditional active methods. Considering the significance of 2D acoustic vortex production and merits of the proposed mechanism in terms of simplicity, efficiency, device size, precision, flexibility and energy consumption, we envision that our scheme will should be a considerable method for 2D acoustic vortex generation and may have far-reaching implication in many diverse fields such as on-chip hologram and particle manipulation.

## Methods

Throughout the paper, the numerical simulations are performed with ‘Acoustic-Solid Interaction Module’ in commercial software COMSOL Multiphysics. The solid material for building the hybrid structure is chosen as brass for which the sound speed and mass density are *c* = 4000 m/s and *ρ* = 8600 kg/m^3^ respectively. The mass density *ρ*_m_, Young’s modulus *E*, and Poisson’s ratio *ν* of the membrane are 1425 kg/m^3^, 1 GPa, and 0.33, respectively. No tension is applied to the membranes and the membranes are modeled as acoustically thin plates. The background medium is air whose density *ρ*_0_ = 1.21 kg/m^3^ and sound speed *c*_0_ = 343 m/s.
